# Identification of Tissue-Specific Expressed Hub Genes and Potential Drugs in Rheumatoid Arthritis Using Bioinformatics Analysis

**DOI:** 10.3389/fgene.2022.855557

**Published:** 2022-03-18

**Authors:** Xuewu Xing, Qun Xia, Baoqi Gong, Zhongyang Shen, Yingze Zhang

**Affiliations:** ^1^ Department of Orthopaedics, Tianjin First Central Hospital, Tianjin, China; ^2^ School of Medicine, Nankai University, Tianjin, China; ^3^ Department of Rheumatology, Tianjin First Central Hospital, Tianjin, China; ^4^ Department of Transplant Surgery, Tianjin First Central Hospital, Tianjin, China; ^5^ Department of Orthopaedic Surgery of Hebei Province, Third Hospital of Hebei Medical University, Shijiazhuang, China; ^6^ Chinese Academy of Engineering, Beijing, China

**Keywords:** rheumatoid arthritis, GEO database, tissue specific, hub gene, competing endogenous RNA

## Abstract

**Background:** Rheumatoid arthritis (RA) is a common autoimmune disease characterized by progressive, destructive polyarthritis. However, the cause and underlying molecular events of RA are not clear. Here, we applied integrated bioinformatics to identify tissue-specific expressed hub genes involved in RA and reveal potential targeted drugs.

**Methods:** Three expression profiles of human microarray datasets involving fibroblast-like synoviocytes (FLS) were downloaded from the Gene Expression Omnibus (GEO) database, the differentially expressed mRNAs (DEGs), miRNAs (DEMs), and lncRNAs (DELs) between normal and RA synovial samples were screened using GEO2R tool. BioGPS was used to identified tissue-specific expressed genes. Functional and pathway enrichment analyses were performed for common DEGs using the DAVID database, and the protein-protein interaction (PPI) network of common DEGs was constructed to recognize hub genes by the STRING database. Based on receiver operating characteristic (ROC) curve, we further investigated the prognostic values of tissue-specific expressed hub genes in RA patients. Connectivity Map (CMap) was run to identify novel anti-RA potential drugs. The DEM–DEG pairs and ceRNA network containing key DEMs were established by Cytoscape.

**Results:** We obtain a total of 418 DEGs, 23 DEMs and 49 DELs. 64 DEGs were verified as tissue-specific expressed genes, most derive from the hematologic/immune system (20/64, 31.25%). GO term and KEGG pathway enrichment analysis showed that DEGs focused primarily on immune-related biological process and NF-κB pathway. 10 hub genes were generated via using MCODE plugin. Among them, SPAG5, CUX2, and THEMIS2 were identified as tissue-specific expressed hub genes, these 3 tissue-specific expressed hub genes have superior diagnostic value in the RA samples compared with osteoarthritis (OA) samples. 5 compounds (troleandomycin, levodopa, trichostatin A, LY-294002, and levamisole) rank among the top five in connectivity score. In addition, 5 miRNAs were identified to be key DEMs, the lncRNA–miRNA–mRNA network with five key DEMs was formed. The networks containing tissue-specific expressed hub genes are as follows: ARAP1-AS2/miR-20b-3p/TRIM3, ARAP1-AS2/miR-30c-3p/FRZB.

**Conclusion:** This study indicates that screening for identify tissue-specific expressed hub genes and ceRNA network in RA using integrated bioinformatics analyses could help us understand the mechanism of development of RA. Besides, SPAG5 and THEMIS2 might be candidate biomarkers for diagnosis of RA. LY-294002, trichostatin A, and troleandomycin may be potential drugs for RA.

## Introduction

Rheumatoid arthritis (RA) is a chronic systemic autoimmune disease characterized by hyperplastic synovium, invasive synovitis, and progressive joint damage (Scott et al., 2010). The incidence of RA is approximately 1% in the adult population, mainly affecting middle-aged to elderly women (Stephenson et al., 2018). RA is the leading cause of disability worldwide and imposes a significant public health and economic burden (Schmidt et al., 2020). The etiology of RA is complex and multifactorial (McInnes and Schett, 2011). Despite there have been major advances in the understanding of RA development, the pathogenesis of RA remains incompletely elucidated.

Microarray analyses has improved the ability of human to study the pathogenesis of diseases, increasingly being used to explore disease epigenetics and to screen for effective biomarkers for disease diagnosis and treatment (Kulasingam and Diamandis, 2008). Although several genes, such as HLA-DR, PTPTN22, CTLA-4, and PADI4, have been identified as genetic factors that contribute to RA susceptibility, we still don’t know the exact mechanisms by which these genes trigger RA (Moran-Moguel et al., 2018). Therefore, we can elucidate the pathogenesis of RA by identifying potential biomarkers and potential therapeutic targets. In addition, the competing endogenous RNA (ceRNA) hypothesis could facilitate the elucidation of molecular mechanisms underlying disease progression (Salmena et al., 2011). Tissue-specific expressed hub genes and regulatory networks of disease may be screened through transcriptome microarray and bioinformatic analysis (Cheng et al., 2021).

Del Rey et al. conducted transcriptional analysis of normal and pathological synoviocytes using microarray expression profiling (Del Rey et al., 2012). Georgel P et al. used high throughput expression analysis to evaluate the overall miRNA expression level in fibroblast-like synoviocytes (FLS) isolated from RA patients in comparison with FLS from osteoarthritic (OA) patients (Liu and Ren, 2020). Bi et al. assessed the differential expression profiles of lncRNA in FLSs between the RA group and the healthy control groups (Bi et al., 2019). In the present study, we downloaded the expression profiles of the above three different types of RNA from GEO database to screen the differentially expressed mRNAs (DEGs), miRNAs (DEMs), and lncRNAs (DELs) between normal and RA synovial samples. Hub genes, tissue-specific expressed genes, RNA regulatory networks, and novel anti-RA potential drugs were obtained by comprehensive analysis. This work provides a deeper understanding of the molecular mechanism underlying RA development and reveals novel biomarkers for disease diagnosis and potential drug for disease therapy.

## Materials and Methods

### Datasets Collection

Three human microarray datasets involving fibroblast-like synoviocytes (FLS) were obtained by searching NCBI’s Gene Expression Omnibus (GEO) database (http://www.ncbi.nlm. nih. gov/geo/). The mRNA, miRNA and lncRNA expression profiles were procured from GSE29746, GSE72564 and GSE128813 respectively, the description of these data sets was detailed below (Table 1).

**TABLE 1:** Details of the 3 human microarray datasets.

	GSE29746	GSE72564	GSE128813
Expression profiling	mRNA	miRNA	lncRNA
Public on	25 Oct 2011	01 Sep 2015	08 Jan 2020
Organization	Spain	France	China
Samples of RA group	9	4	3
Samples of control group	11	4	3
Platforms	GPL4133 Agilent	GPL20870 Qiagen	GPL21827 Agilent

RA, rheumatoid arthritis; OA, osteoarthritis.

### Authentication of DEG, DEM, and DEL

Through GEO2R online tool, we obtained differentially expressed mRNAs (DEGs), miRNAs (DEMs) and lncRNAs (DELs). GEO2R is an interactive online tool that can be used to compare multiple expression data and then identify differentially expressed profiles. We selected differentially expressed RNAs according to the following criteria: *p* < 0.05 and |log2 (fold change) |≥1.

### Authentication of the Tissue-specific Expressed Genes

We analyzed the distribution of DEGs using an online tool BioGPS (http://biogps.org/) which was created as a centralized gene-annotation portal for clustering distributed gene (Wu et al., 2009). Tissue-specific genes should qualify the following criteria: 1) the expression level of transcripts that mapped to a single organ system was >10 times the median, and 2) the highest expression level was more than half as high as the second highest level (Cheng et al., 2021).

### Functional Enrichment Analysis

To identify the function of DEGs, the enrichment analysis was conducted by DAVID (version 6.8) using Gene Ontology (GO) and Kyoto Encyclopedia of Genes and Genomes (KEGG) pathways database (Huang et al., 2007). We defined enriched functions and pathways using a cutoff of *p* < 0.05.

### Construction and Analysis of Protein–Protein Interaction (PPI) Network

A PPI network was constructed for the DEGs using STRING (https://string-db.org/) with a filter condition (interaction score>0.4), a online tool of known, and predicted PPIs. We use the Cytoscape to visualize the network. In order to screen the important pathways related to the development of RA, we constructed an interaction network between DEGs and the signaling pathway using the ClueGO plug-in of Cytoscape platform. We applied MCODE plug-in of Cytoscape to identify hub genes of significant modules with following criteria: MCODE scores >5, degree cut-off = 2, node score cut-off = 0.2, Max depth = 100, and k-score = 2.

### CMap Analysis

We run Connectivity Map (CMap) to identify novel anti-RA potential drugs. Through the database which is available online (http://www.broadinstitute.org/cmap/), we can get the similarity between each researcher’s DEGs and 7,056 gene expression profiles caused by 1,309 bioactive compounds (Huang et al., 2007). The similarity is assessed by the ‘connectivity score’. The connectivity score ranges from −1 to +1; a positive score means a promoted effect, while a negative score means an inhibited effect (Peng et al., 2020).

### Construction of kDEM-DEG Pairs

The potential microRNA binding to DEGs were predicted using TargetScan (http://www.targetscan.org/vert_72/). The key differentially expressed miRNAs (kDEM) were obtained by the intersection between predicted miRNAs and differentially expressed miRNAs found from the GSE72564 dataset. The kDEM–DEG pairs were constructed using Cytoscape.

### Establishment of the ceRNA Network

The miRcode database (http://www.mircode.org/) was used to predict miRNA that can interact with DELs. We used Cytoscape to construct the ceRNA network by selecting lncRNA–miRNA–mRNA interaction that contain key DEMs.

## Results

### Identification of DEGs

**FIGURE 1:**
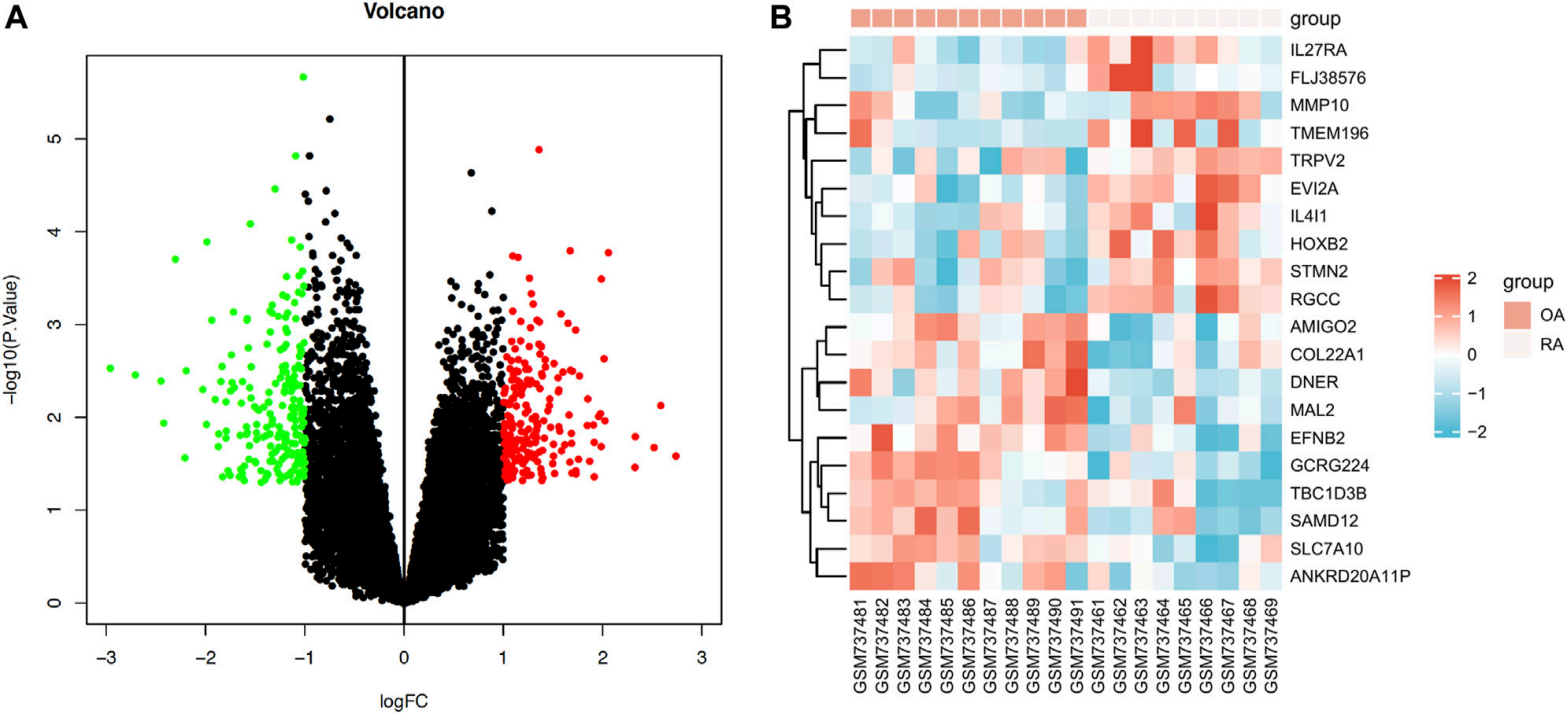
Expression profiles of differentially expressed mRNAs (DEGs) in RA. **(A)** Volcano plot of DEGs (red: upregulated DEGs. green: downregulated DEGs.) **(B)** Heatmap of the top 10 upregulated DEGs and the top 10 downregulated DEGs (red: high expression; blue: low expression).

We reanalyzed the GEO data of GSE29746 in order to compare the expression pattern of differential genes between pathological synovial tissues from 9 rheumatoid arthritis (RA) patients and normal synovial tissues from 11 sex and age matched osteoarthritis (OA) patients. A total of 418 DEGs were obtained, comprising 206 upregulated genes and 212 downregulated genes. The data of DEGs was visualized as a volcano plot (Figure 1A), The top 10 DEGs were displayed in a heatmap (Figure 1B).

### Authentication of the tissue-Specific Expressed Genes

We identified 64 tissue-specific expressed genes using BioGPS (Table 2). The system with the greatest distribution of tissue-specific expressed genes is the hematologic/immune system (20/46, 31.25%), followed by the digestive system (11), nervous system (10), endocrine system (7), genital system (6), respiratory system (6), urinary system (2), circulatory system (1) and motor system (1).

**TABLE 2:** Distribution of tissue specific expressed genes identified by BioGPS.

system (counts)	Tissue	Genes
Hematologic/Immune (20)	CD56 + NK cells	TRPV2, PTGER2, ADGRG1, PREX1, NKG7
	BDCA4+dendritic cells	LAMP5, CUX2
	CD19 + B cells	VPREB3, BIRC3
	CD14 + monocytes	ADGRE1, TNFAIP8L2
	CD33 + myeloid	CCR1, TLR4, THEMIS2
	721 B lymphoblasts	IFI44L, SPAG5
	CD4+T cells	TRABD2A
	CD8+T cells	TBC1D10C
	whole blood	CLEC2B, KCNJ15
Digestive (11)	colorectal adenocarcinoma	HOXB7, MSX1, NGFR, KRT80, RBP1
	salivary gland	CST2, OPRPN
	small intestine	SLC5A1
	liver	HRG, HAAO, PLG
Nervous (10)	fetal brain	STMN2, LDLRAD4, DLX1
	whole brain	CRYM, BRINP1, MAPRE3
	prefrontal cortex	SHC3, CERCAM
	retina	FRZB, AOC2
Endocrine (7)	pineal	BEX5, CDH10, IGSF21, ASMT
	pancreatic islet	CRP
	thyroid	DUOX2, ID4
Genital (6)	testis germ cells	CHODL
	testis intersitial	SPATA8, LYZL6
	uterus	OGN
	uterus corpus	LEFTY2, MMP10
Respiratory (6)	lung	NEDD9, SFTPB, HEY1
	bronchial epithelial cells	PHLDA1, AMIGO2, FZD6
Urinary (2)	kidney	FMO1, CRYAA
Circulatory (1)	heart	HHATL
Motor (1)	skeletal muscle	ACTN3

CD, cluster differentiation.

### GO Term and KEGG Pathway Enrichment Analysis of DEGs

**FIGURE 2:**
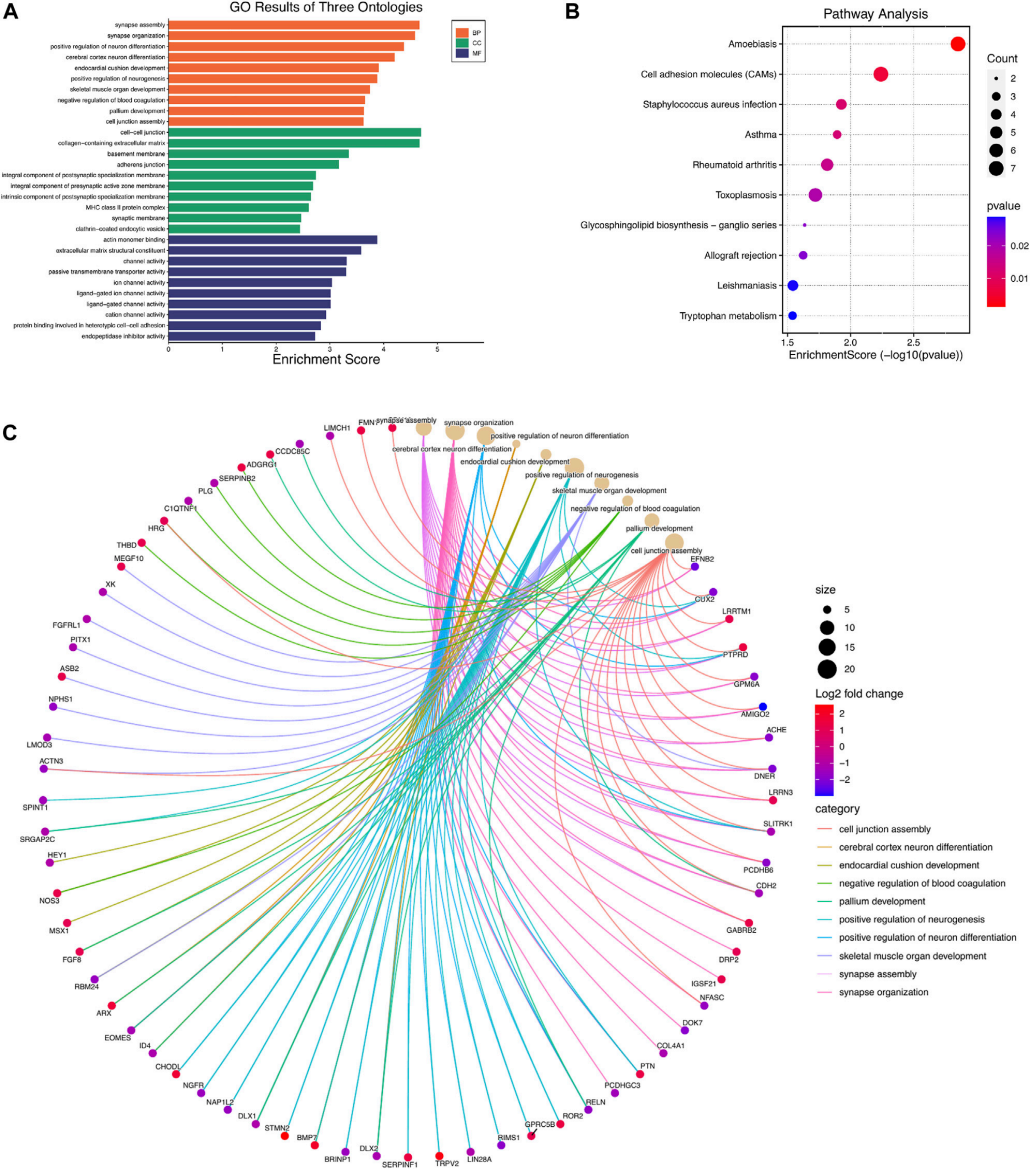
GO and KEGG pathway enrichment analyses of differentially expressed mRNAs (DEGs). **(A)** Top 10 enriched GO terms of the DEGs. **(B)** cnetplot showing the top 10 enriched biological processes of DEGs **(C)**. The bubble plot showing top 10 enriched KEGG pathways of DEGs.

GO analysis showed that the biological processes (BP) of DEGs focused primarily on synapse assembly, synapse organization, positive regulation of neuron differentiation, cerebral cortex neuron differentiation, endocardial cushion development, positive regulation of neurogenesis, skeletal muscle organ development, negative regulation of blood coagulation, pallium development, and cell junction assembly (Figures 2A,B). The main cellular components (CC) include cell-cell junction, collagen-containing extracellular matrix, basement membrane, adherent junction, integral component of postsynaptic specialization membrane, integral component of presynaptic active zone membrane, intrinsic component of postsynaptic specialization membrane, MHC class II protein complex, synaptic membrane, and clathrin-coated endocytic vesicle (Figure 2A). The molecular functions (MF) include actin monomer binding, extracellular matrix structural constituent, channel activity, passive transmembrane transporter activity, ion channel activity, ligand-gated ion channel activity, ligand-gated channel activity, cation channel activity, protein binding involved in heterotypic cell-cell adhesion, and endopeptidase inhibitor activity (Figure 2A). Genes are mainly involved in the KEGG pathway termed Amoebiasis, Cell adhesion molecules (CAMs), Staphylococcus aureus infection, Asthma, Rheumatoid arthritis, Toxoplasmosis, Glycosphingolipid biosynthesis-ganglio series, Allograft rejection, Leishmaniasis, and Tryptophan metabolism (Figure 2C; Table 3).

**TABLE 3:** Enrichment analysis of KEGG pathway.

KEGG ID	Term	GeneRatio	PValue	Genes
hsa05146	Amoebiasis	7/94	0.001414621	ACTN3/LAMA1/GNA14/TLR4/COL4A1/SERPINB4/SERPINB2
hsa04514	Cell adhesion molecules (CAMs)	7/94	0.005757522	HLA-DRB5/NCAM2/HLA-DQA2/HLA-DRB4/NFASC/CDH15/CDH2
hsa05150	Staphylococcus aureus infection	4/94	0.011840374	HLA-DRB5/HLA-DQA2/HLA-DRB4/PLG
hsa05310	Asthma	3/94	0.012784974	HLA-DRB5/HLA-DQA2/HLA-DRB4
hsa05323	Rheumatoid arthritis	5/94	0.015329475	HLA-DRB5/TLR4/HLA-DQA2/ATP6V1C2/HLA-DRB4
hsa05145	Toxoplasmosis	6/94	0.019005793	LAMA1/HLA-DRB5/BIRC3/TLR4/HLA-DQA2/HLA-DRB4
hsa00604	Glycosphingolipid biosynthesis - ganglio series	2/94	0.023088868	ST6GALNAC3/ST6GALNAC5
hsa05330	Allograft rejection	3/94	0.023727967	HLA-DRB5/HLA-DQA2/HLA-DRB4
hsa05140	Leishmaniasis	4/94	0.028621472	HLA-DRB5/TLR4/HLA-DQA2/HLA-DRB4
hsa00380	Tryptophan metabolism	3/94	0.028807929	ASMT/IL4I1/HAAO

KEGG , kyoto encyclopedia of genes and genomes.

### Construction and Analysis of PPI Network

**FIGURE 3:**
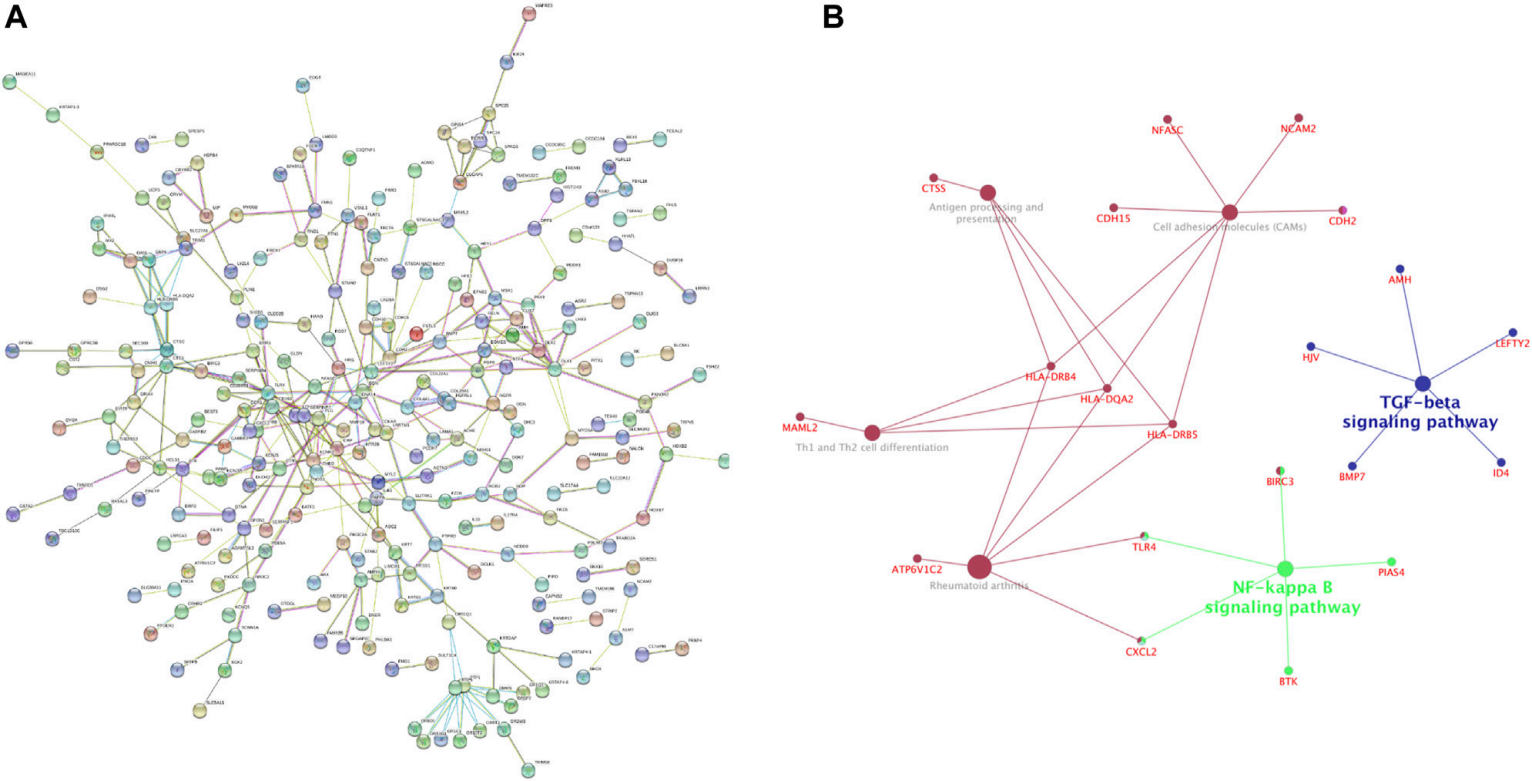
Construction and analysis of protein-protein interaction (PPI) network **(A)** PPI network of differentially expressed mRNAs (DEGs) constructed with STRING databases **(B)** Gene-pathway network related to the development of Rheumatoid arthritis (RA).

Based on the identified DEGs and the STRING database, we constructed a PPI network containing 364 nodes and 361 edges (Figure 3A). We found out that NF-κB pathway may be involved in the pathogenesis of RA through Gene-pathway network constructed by The ClueGO plugin of Cytoscape (Figure 3B). A total of 13 important module were generated via using MCODE plugin in Cytoscape, and 10 hub genes were obtained (Table 4).

**TABLE 4:** Hub gene obtained by MCODE plug-in of Cytoscape.

No	Gene symbol	Log FC	*p* Value	Full name
1	MMP10	2.739087238	0.026051857	matrix metallopeptidase 10
2	TRIM3	−1.3827721	0.03985551	tripartite motif containing 3
3	THEMIS2	1.24876953	0.0083544	thymocyte selection associated family member 2
4	DLX2	−1.1895073	0.01692604	distal-less homeobox 2
5	SPAG5	1.03248394	0.0068909	sperm associated antigen 5
6	MIP	−1.1818731	0.00050311	major intrinsic protein of lens fiber
7	ASB2	1.20540696	0.0045946	ankyrin repeat and SOCS box containing 2
8	CXCL2	1.18019999	0.01749176	C-X-C motif chemokine ligand 2
9	CUX2	−1.9375622	0.00088542	cut like homeobox 2
10	FRZB	−1.5413208	0.00284952	frizzled-related protein

SOCS, suppressor of cytokine signalling.

### ROC Curve of the 3 Tissue-Specific Expressed Hub Genes

**FIGURE 4:**
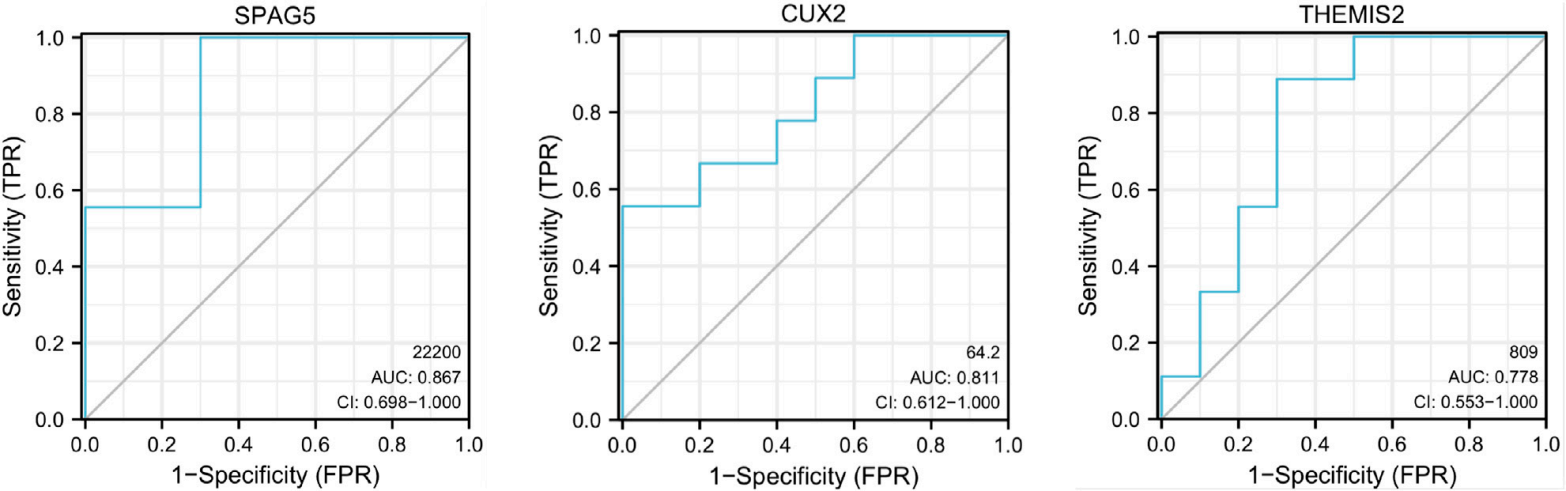
ROC curve of the 3 specifically expressed hub genes in Rheumatoid arthritis (RA) samples. AUC area under the ROC curve.

Furthermore, we analyzed the performance of 3 tissue-specific expressed hub genes in diagnosing RA by means of receiver operating characteristic (ROC) curve analysis in SPSS 23.0 software. The area under the ROC curve (AUC) was calculated to indicate diagnostic efficiency and predictive accuracy (Cheng et al., 2021). These 3 tissue-specific expressed hub genes have superior diagnostic value in the RA samples compared with OA samples. Specifically, SPAG5 showed the highest diagnostic performance (AUC: 0.867) in the RA samples, closely followed by CUX2 (AUC: 0.811), THEMIS2 ranks last (AUC: 0.778) (Figure 4). Based on the above data, these 3 tissue-specific expressed hub genes may serve as potential biomarkers for RA diagnosis.

### Identification of Potential Targeted Drugs by CMap Analysis

We obtained a list of compounds after uploading DEGs tags to the CMap database. The top five compounds with the highest scores which may be potential drugs for RA are shown in Table 5.

**TABLE 5:** The top five compounds with the highest scores in the CMap analysis.

CMap name	Dose	Cell line	Score	Instance ID	Description
Troleandomycin	5 µM	HL60	−1	1,965	inhibitors of CYP450 monooxygenases
Levodopa	20 µM	HL60	−0.998	1,972	the levorotatory form of dopa used in Parkinson’s disease
Trichostatin A	100 nM	HL60	−0.971	2,949	HDAC inhibitor
LY-294002	10 µM	MCF7	−0.952	1,074	MTOR inhibitor
Levamisole	17 µM	HL60	−0.951	1,410	an anthelmintic drug C_11_H_12_N_2_S

CYP450, Cytochrome P450; HDAC, histone deacetylase; MTOR, mammalian target of rapamycin.

### Identification of DEMs and Construction of DEM-DEG Pairs

**FIGURE 5:**
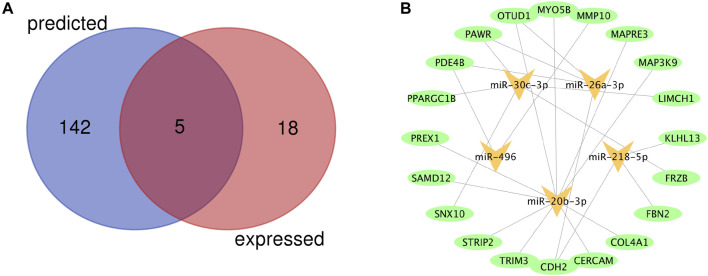
Identification of key differentially expressed miRNAs (DEMs) and construction of DEM-DEG Pairs **(A)** Venn diagram of key differentially expressed miRNAs between predicted miRNAs and DEMs. **(B)** The kDEM–DEG pairs constructed by Cytoscape.

A total of 23 miRNAs were identified as DEMs, of which 17 miRNAs were upregulated and 6 miRNAs were downregulated (Table 6). A total of 147 miRNAs were predicted to combine with the DEGs. A total of five key DEMs were procured (Figure 5A), of which four were up-regulated, namely, miR-30c-2-3p, miR-20b-3p, miR-26a-1-3p, miR-218-5p, and miR-496 was the only down-regulated one. The kDEM–DEG pair is shown in Figure 5B.

**TABLE 6:** differentially expressed miRNAs (DEMs) obtained from the GSE72564.

ID	adj.P.Val	P.Value	t	B	logFC	miRNA_ID
846	0.773	0.00181	5.08274	−0.98	1.5375	miR-670-5p
626	0.773	0.00365	4.44126	−1.51	2.0325	miR-26a-1-3p
958	0.773	0.00745	3.83781	−2.08	1.895	miR-2116-5p
63	0.773	0.02222	2.99059	−2.99	1.1625	miR-190a-5p
101	0.773	0.02336	2.95362	−3.03	1.5025	miR-548b-3p
119	0.773	0.02551	2.88896	−3.11	1.285	miR-579-3p
387	0.773	0.02961	2.78028	−3.24	1.7775	miR-1305
408	0.773	0.02995	2.77201	−3.25	1.6525	miR-30c-3p
620	0.773	0.03323	2.69675	−3.34	1.115	miR-20b-3p
797	0.773	0.03394	2.68158	−3.35	1.185	miR-4262
267	0.773	0.0345	2.66977	−3.37	1.165	miR-502-3p
666	0.773	0.0354	2.65136	−3.39	1.425	miR-1258
508	0.773	0.03672	2.62497	−3.42	2.1575	miR-708-5p
479	0.773	0.04256	2.51956	−3.55	1.6225	miR-1299
284	0.773	0.04671	2.45336	−3.63	2.745	miR-218-5p
731	0.773	0.04784	2.43638	−3.65	1.1275	miR-3116
636	0.773	0.04941	2.41358	−3.68	1.4275	miR-499a-5p
1	0.773	0.0471	−2.44742	−3.64	−1.02	miR-346
813	0.773	0.04084	−2.54889	−3.51	−1.475	miR-1193
1,067	0.773	0.03396	−2.68116	−3.35	−1.045	miR-4263
264	0.773	0.028	−2.82107	−3.19	−1.0725	miR-496
889	0.773	0.02301	−2.96491	−3.02	−1.8875	miR-2276-3p
560	0.773	0.01371	−3.35482	−2.58	−1.2775	miR-653-5p

DEG, differentially expressed mRNAs; DEM, differentially expressed miRNAs; DEL, differentially expressed lncRNAs; miR, miRNA; FC, fold change.

### Identification of DELs and Establishment of the ceRNA Network

**FIGURE 6:**
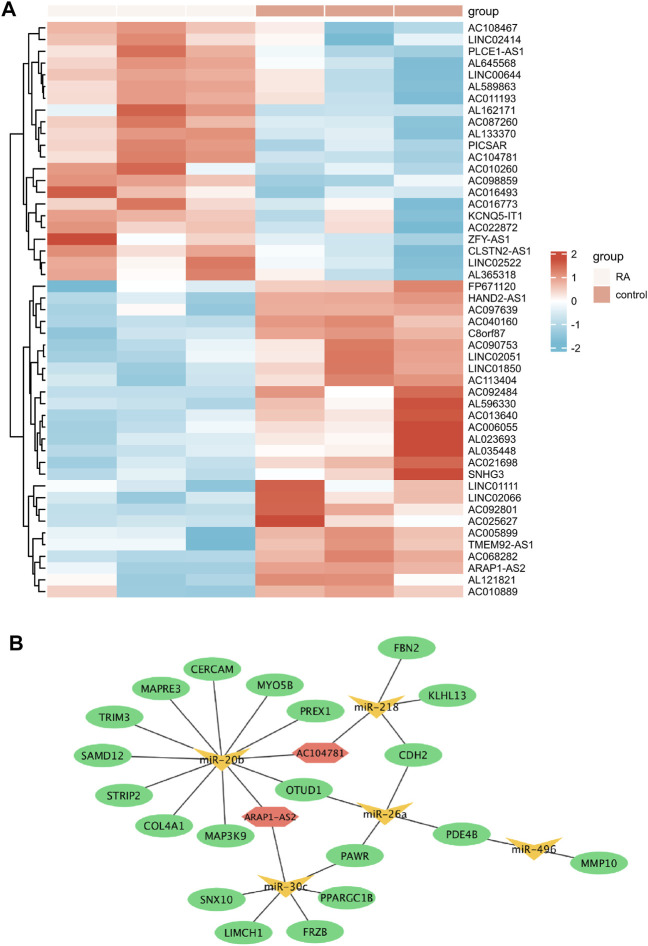
Identification of differentially expressed lncRNAs (DELs) and Establishment of the ceRNA network **(A)** Heatmap of the DELs (red: high expression; blue: low expression) **(B)** The ceRNA network in Rheumatoid arthritis (RA) (ellipse: mRNA, triangle: miRNA, and hexagonal: lncRNA).

A total of 49 DELs were identified, of which 22 were up-regulated and 27 were down-regulated in GSE128813 (Figure 6A). The interactive miRNAs of DELs were predicted by mircode. Among these 49 lncRNAs, ARAP1-AS2, and AC104781 interacted with the five key miRNAs. ceRNA network was constructed by Cytoscape (Figure 6B). There are two networks containing hub gene: ARAP1-AS2/miR-20b-3p/TRIM3, ARAP1-AS2/miR-30c-3p/FRZB.

## Discussion

RA is one of the most common chronic autoimmune disease and is heterogeneous with complex pathogeneses. Although there is an accumulating evidence suggesting the involvement of novel transcripts in the development of RA, detailed mechanisms still remain elusive. In this study, we obtained the 418 DEGs in FLS from RA patients and OA patients. We further perform a tissue-specific expression analysis of DEGs, the result reveals that the hematologic/immune system is the greatest distribution system, which may explain the disease occurrence of RA.

Module analysis of the PPI network using ClueGO uncovered that NF-κB pathway may perform a significant role in RA pathogenesis. This has been confirmed by a number of studies. B cell activating factor of TNF family (BAFF) promoted synovial inflammation by activating of B cells in RA through NF-κB signaling pathway (Zhang et al., 2021). Growth differentiation factor 11 (GDF11) was able to significantly restrain the nuclear factor kappa-light-chain-enhancer of activated B cells (NF-κB) signaling pathway and prevent the development of RA (Li et al., 2019). Overexpression of miR-496 in RA-FLS may inhibit cell proliferation by inactivating the NF-κB pathway (Xing et al., 2021). CS-semi5, a semisynthetic chondroitin sulfate, may effectively improve synovial inflammation, and cartilage erosion in RA through NF-κB deactivation (Li et al., 2021). Hence, the intrinsic relationship between NF-κB pathway and RA needs to be further studied in the future.

Module analysis of the PPI network using MCODE identified 3 tissue-specific expressed hub genes involving in the hematologic/immune system. SPAG5 is a Protein Coding gene involved in the functional and dynamic regulation of mitotic spindles. There is growing evidence that it is upregulated in many human cancers, acting as an oncogene and promoting cell proliferation (Liu et al., 2018; He et al., 2020; Canu et al., 2021). CUX2 is a Protein Coding gene encoding a transcription factor which contains three CUT domains and a homeodomain. CUX2 functions as an accessory factor in the repair of oxidative DNA damage (Pal et al., 2015) and plays an important role in the neuron specification and spine development (Cubelos et al., 2010). THEMIS2, also known as ICB1 (Induced on contact with basement membrane 1), is a protein expressed in B cells and is associated with signaling proteins Grb2 and Vav1 (Hartweger et al., 2014). Themis2 is not required for B cell development and activation, but it lowers the threshold for activation of B cells in positive selection (Cheng et al., 2017). Themis2 may constitute a control point in macrophage inflammatory response (Peirce et al., 2010). There have been no reports of three tissue-specific expressed hub genes in RA-related research. Nonetheless, SPAG5 and THEMIS2 were overexpressed in the synovial samples of RA in our study. In addition, the results of ROC analysis revealed that these genes had an important diagnostic value for RA. Therefore, these findings suggest that SPAG5 and THEMIS2 may play an essential in progression of synovial hyperplasia.

After interacting 147 DEGs-related target miRNAs and 23 DEMs, 5 overlapping miRNAs were obtained as key miRNAs for further study. In addition to miR-20b and miR-30c, the roles of the other three miRNAs in RA have been reported. miR-496 can impair the proliferative ability and facilitate the apoptosis of IL-1β-treated MH7A through regulating MMP10 expression and NF-κB signaling pathway (Xing et al., 2021). miR-218-5p knockdown could modulate the proliferation, apoptosis and autophagy of RASFs by upregulating KLF9, and suppressing the activation of the JAK2/STAT3 signaling pathway (Chen et al., 2021). MiR-218 regulates the osteogenic differentiation of RA-FLS via the ROBO1/DKK-1 axis (Iwamoto et al., 2018). miR-26a could enhances cells proliferation and attenuate apoptosis of chondrocytes in RA rats (Jiang and Cao, 2020). In addition, miR-26a ameliorates the arthritis severity of the rats through directly targeting TLR3 in rat macrophages (Jiang et al., 2014). Given its prominent role in RA, plasma miR-26a has been proposed as the most promising non-invasive biomarkers for disease detection (Liu et al., 2019). Although there is no relevant research on miR-20b and miR-30c in RA, studies have shown that both of them have a definite effect on inflammation. miR-30c-5p adjusts macrophage-mediated inflammation through pro-apoptotic signals (Ceolotto et al., 2017). knockdown of lncRNA HOTAIR inhibits inflammatory cytokine secretion by increasing the expression of miR-20b and decreasing the expression of NLRP3, thereby relieving ankle swelling caused by gouty arthritis (Liu et al., 2021). miR-20b can restraint T Cell proliferation and activation by regulating NFAT Signaling Pathway in Thymoma-Associated Myasthenia Gravis (Xin et al., 2016). However, given that these results are based only on bioinformatics models and literature reports, further research is essential to validate the in-depth role of these 5 key miRNAs in RA.

We establish a ceRNA regulatory network was constructed based on five key miRNAs to investigate the role of lncRNA in RA. There are two regulatory axes involving hub genes among the network, including ARAP1-AS2/miR-20b-3p/TRIM3, ARAP1-AS2/miR-30c-3p/FRZB. Interestingly, the key miRNAs involved in these two regulatory networks are the same ones that have not been reported for RA. TRIM3 and FRZB are hub genes involved in the regulation of these two ceRNA axes, both are down regulated in our study. It is worth noting that our finding of FRZB differ from a prior study on circulating levels of FRZB in patients with early rheumatoid arthritis (Corallini et al., 2010). Experimental study has shown that TRIM3 expression in synovial tissue samples from patients with RA was lower than that of healthy controls, which is consistent with our findings, may play an anti-proliferative role in RA-FLS through the P38 signaling pathway (Wang et al., 2017). Our findings also offer insights into the roles of ARAP1-AS2. Unlike for rheumatoid arthritis, the study by Yang et al. showed that lncRNA-ARAP1-AS2 gradually up-regulated with the progression of diabetes (Yang et al., 2019). Mechanistically, lncRNA ARAP1-AS2 may participate in high glucose-induced proximal tubular cell injury by interacting with ARAP1 through persistent activation of EGFR/TGF-β/Smad3 pathway (Li X et al., 2020). ARAP1-AS2/ARAP1 may affect EMT processes and cytoskeleton rearrangement in human renal tubular epithelial cells via boosting of Cdc42-GTP levels (Li L et al., 2020). Although lncRNA ARAP1-AS2 has not been reported in RA, we assumed that ARAP1-AS2/miR-20b-3p/TRIM3 or ARAP1-AS2/miR-30c-3p/FRZB might be the key regulatory axes in the pathogenesis of RA based on the above analysis.

According to CMap analysis, five potential compounds for RA treatment were obtained, and their targets, and application scope were further searched. Troleandomycin (TAO) is one of cytochrome P-450 (CYP450) monooxygenases blockers, which is considered to be a novel drug in the treatment of human inflammatory bowel disease (Sasaki et al., 2003). This suggests that its therapeutic role in RA is also worth exploring. Antirheumatic agents are effective for Parkinson’s symptoms, but not for levodopa (Lee et al., 2020). Nonselective HDAC inhibitor trichostatin A (TSA) shared multiple molecular mechanism in suppressing inflammation and may represent a new principle in the treatment of RA (Chung et al., 2003; Gillespie et al., 2012; Grabiec et al., 2012). As a PI3K/AKT inhibitor, LY294002 is often used in RA-related studies (Brennan et al., 2002; Kim et al., 2005; Xing et al., 2016). This presents a number of possibilities for therapeutic strategies. Levamisole was previously used to treat rheumatoid arthritis due to its immunomodulatory properties. However, it had been withdrawn from the US market because of serious side-effects (Cascio and Jen, 2018).

## Conclusion

This study indicates that screening for identify tissue-specific expressed hub genes and ceRNA network in RA using integrated bioinformatics analyses could help us understand the mechanism of development of RA. Besides, SPAG5 and THEMIS2 might be candidate biomarkers for diagnosis of RA. LY-294002, trichostatin A, and troleandomycin may be potential drugs for RA.

## Data Availability

The original contributions presented in the study are included in the article/Supplementary Material, further inquiries can be directed to the corresponding author.
